# Effect of Sodium-Glucose Co-Transporter 2 Inhibitor, Dapagliflozin, on Renal Renin-Angiotensin System in an Animal Model of Type 2 Diabetes

**DOI:** 10.1371/journal.pone.0165703

**Published:** 2016-11-01

**Authors:** Seok Joon Shin, Sungjin Chung, Soo Jung Kim, Eun-Mi Lee, Young-Hye Yoo, Ji-Won Kim, Yu-Bae Ahn, Eun-Sook Kim, Sung-Dae Moon, Myung-Jun Kim, Seung-Hyun Ko

**Affiliations:** 1 Division of Nephrology, Department of Internal Medicine, College of Medicine, The Catholic University of Korea, Seoul, Korea; 2 Division of Endocrinology & Metabolism, Department of Internal Medicine, College of Medicine, The Catholic University of Korea, Seoul, Korea; 3 Department of Physiology, College of Medicine, The Catholic University of Korea, Seoul, Korea; CHA University, REPUBLIC OF KOREA

## Abstract

**Background:**

Renal renin-angiotensin system (RAS) activation is one of the important pathogenic mechanisms in the development of diabetic nephropathy in type 2 diabetes. The aim of this study was to investigate the effects of a sodium-glucose co-transporter 2 (SGLT-2) inhibitor, dapagliflozin, on renal RAS in an animal model with type 2 diabetes.

**Methods:**

Dapagliflozin (1.0 mg/kg, OL-DA) or voglibose (0.6 mg/kg, OL-VO, diabetic control) (n = 10 each) was administered to Otsuka Long-Evans Tokushima Fatty (OLETF) rats for 12 weeks. We used voglibose, an alpha-glucosidase inhibitor, as a comparable counterpart to SGLT2 inhibitor because of its postprandial glucose-lowering effect without proven renoprotective effects. Control Long-Evans Tokushima Otsuka (LT) and OLETF (OL-C) rats received saline (n = 10, each). Changes in blood glucose, urine albumin, creatinine clearance, and oxidative stress were measured. Inflammatory cell infiltration, mesangial widening, and interstitial fibrosis in the kidney were evaluated by histological analysis. The effects of dapagliflozin on renal expression of the RAS components were evaluated by quantitative RT-PCR in renal tissue.

**Results:**

After treatment, hyperglycemia and urine microalbumin levels were attenuated in both OL-DA and OL-VO rather than in the OL-C group (*P* < 0.05). The urine angiotensin II (Ang II) and angiotensinogen levels were significantly decreased following treatment with dapagliflozin or voglibose, but suppression of urine Ang II level was more prominent in the OL-DA than the OL-VO group (*P* < 0.05). The expressions of angiotensin type 1 receptor and tissue oxidative stress markers were markedly increased in OL-C rats, which were reversed by dapagliflozin or voglibose (*P* < 0.05, both). Inflammatory cell infiltration, mesangial widening, interstitial fibrosis, and total collagen content were significantly increased in OL-C rats, which were attenuated in OL-DA group (*P* < 0.05).

**Conclusion:**

Dapagliflozin treatment showed beneficial effects on diabetic nephropathy, which might be via suppression of renal RAS component expression, oxidative stress and interstitial fibrosis in OLETF rats. We suggest that, in addition to control of hyperglycemia, partial suppression of renal RAS with an SGLT2 inhibitor would be a promising strategy for the prevention of treatment of diabetic nephropathy.

## Introduction

Diabetic nephropathy is the most common cause of end-stage renal disease (ESRD) in the world. The prevalence of renal complications in patients with type 2 diabetes reaches about 40% with significant progression to ESRD [1–3]. Treatment options have increased substantially over the last decade, but have not yet translated into a remarkable reduction in the incidence of ESRD related to diabetic nephropathy [4]. Therefore, there is an urgent need to identify the agents that have specific effects on the renal complications associated with type 2 diabetes.

Histologically, diabetic nephropathy is characterized by a thickening of the glomerular basement membrane and mesangial matrix expansion, overproduction of hyperglycemia-induced extracellular matrix proteins, and tubulointerstitial fibrosis [5]. Hyperglycemia-induced metabolism, hemodynamic stimuli, oxidative stress, and inflammation are mediators of kidney injury in type 2 diabetes [6]. Among them, the renin-angiotensin system (RAS) is known as an important factor in the development of diabetic nephropathy.

Angiotensin II (Ang II) is a pivotal mediator of RAS and works via activating angiotensin type 1 (AT1R) and type 2 (AT2R) receptors. Activation of AT1R promotes cell growth, and induces vasoconstriction, anti-natriuresis, and an increase in blood pressure [7, 8]. In contrast, AT2R activation inhibits cell growth, promotes cell apoptosis and differentiation, contributes to natriuresis, vasorelaxation, and potentially lowers blood pressure [7–9]. Tissue specific RAS activation in kidneys has been shown to be an important mechanism of renal fibrosis or the progression of diabetic nephropathy [10, 11]. Accumulated evidence has indicated that intrarenal or local RAS in patients with type 2 diabetes is inappropriately activated, leading to local Ang II over-production in glomerular epithelial cells, mesangial cells, and proximal tubular epithelial cells, despite no change or suppression of systemic RAS [12–15]. Our previous report demonstrated that it was not systemic but local RAS activation that induced renal damage associated with local oxidative stress, and intra-renal RAS was activated by high glucose or lipid concentration, inflammatory cytokines or hypoxia [10].

In recent years, sodium glucose co-transporter 2 (SGLT2) inhibitors, which stimulate glucose excretion in the urine, have been proposed as a novel hypoglycemic agent for treating type 2 diabetes [16, 17]. SGLTs are a family of glucose transporters that mediate an active sodium-linked transport process against an electrochemical gradient [18]. In rats and mice, SGLT2 is expressed almost exclusively in the early portion of the proximal convoluted tubule, which is responsible for the majority of glucose reabsorption by the kidney [19]. If SGLT2 inhibitors attenuate renal glucose reabsorption in renal tubules, glucotoxicity in tubular cells and glucose-mediated local RAS activation in the kidney would be attenuated in type 2 diabetes. Therefore, it is anticipated that SGLT2 inhibitors would reduce glucose-mediated renal inflammation and fibrosis. If this is correct, in addition to the glucose-lowering effects, SGLT2 inhibitors might have beneficial effects on the prevention of diabetic nephropathy.

Dapagliflozin is the first-in-class SGLT2 inhibitor, and many clinical studies have shown its effects on glycemic control with both monotherapy and combination therapy with other hypoglycemic agents [20, 21]. Furthermore, dapagliflozin has additional non-glycemic effects including the lowering of blood pressure and a decrease in body weight in several clinical studies [22]. Although some studies using diabetic animal models demonstrated that long-term treatment with SGLT2 inhibitors improved glucose homeostasis and maintained pancreatic beta cell function [23–25], the beneficial effects of dapagliflozin on the renal RAS activation in an animal model of type 2 diabetes with diabetic nephropathy have not been determined.

Voglibose, an alpha-glucosidase inhibitor, inhibits the breakdown of disaccharides into monosaccharides by acting competitively on the activities of an alpha-glucosidase, controlling postprandial hyperglycemia [26, 27]. We used voglibose as a comparable counterpart drug to SLGT2 inhibitor because of its postprandial glucose-lowering effect without hypoglycemia and no proven renoprotective effects.

In this study, we investigated the effect of dapagliflozin on RAS components expression, and whether it could improve diabetic nephropathy or renal injury in diabetic kidney using an animal model with type 2 diabetes.

## Materials and Methods

### Experimental animals

The Otsuka Long-Evans Tokushima Fatty (OLETF) rats are a well-known animal model of human type 2 diabetes, accompanied by characteristic features of obesity, hyperglycemia, hyperlipidemia, and diabetic complications such as diabetic nephropathy [28, 29]. Male 6-week-old OLETF and Long-Evans Tokushima Otsuka (LETO) rats, purchased from Central Lab. Animal Inc. (Seoul, South Korea), were used for this experiment. All rats were housed in a controlled temperature and 12-hour light-dark cycle environment, and supplied with regular rat chow and tap water ad libitum during the period of the whole experiment. Dapagliflozin (AstraZeneca, Mölndal, Sweden) or voglibose (Sigma-Aldrich, St. Louis, MO, USA), as a diabetic control group, was given to OLETF rats by dissolving it in drinking water. Dapagliflozin (1.0 mg/kg, OL-DA, n = 10) or voglibose (0.6 mg/kg, OL-VO, n = 10) was administered, starting at 14 weeks of age.

LETO (non-diabetic control group, LT, n = 10) and OLETF (diabetic control group, OL-C, n = 10) rats received saline. After 12 weeks of treatment, all rats were euthanized and the kidneys removed. Each kidney was divided into the cortex and medulla. All experiments including immunoblots were performed with only the renal cortex. The kidneys were rapidly dissected and stored in 10% buffered formalin for subsequent immunohistochemical analyses. Blood was obtained from the left ventricle and stored at –70°C for subsequent analyses. All of the experimental procedures were approved by the Institutional Animal Care and Use Committee of the Catholic University of Korea.

### Biochemical measurements

Body weight was measured every week. Fasting blood glucose levels were measured with an Accu-check glucometer (Roche, Basel, Switzerland) once per week. The serum creatinine was measured by an autoanalyzer (Hitachi 917, Tokyo, Japan) using commercial kits (Wako, Osaka, Japan). The intraperitoneal glucose tolerance test (2 g/kg of glucose) and hemoglobin A1c (HbA1c), determined by DCA™ STSTEM, HbA1c (Siemens Healthcare Diagnostics Inc. Tarrytown, NY) were performed at 12 weeks of treatment before euthanasia and after fasting overnight. Plasma renin activity (SRL, Tokyo, Japan) and serum aldosterone levels (Diagnostic Systems Laboratories, Webster, TX) were determined using an RIA kit. To measure the 24-h urine volume, urinary albumin excretion, urine chemistry, and creatinine clearance at 12 weeks of treatment, the rats were housed in individual rat metabolic cages (Tecniplast, Gazzada, Italy). Urine glucose and creatinine were determined using an autoanalyzer (Beckman Instruments, Fullerton, CA). The 24-h urinary albumin excretion was measured by immunoassay (Bayer, Elkhart, IN). The creatinine clearance was calculated by (urine [Cr] × urine volume)/(plasma [Cr] × time). To evaluate intrarenal RAS activation, we measured 24-h urinary angiotensinogen (ANG) (Immuno-Biological Laboratories Co., Ltd., Fujioka-shi, Japan) and 24-h urinary Ang II concentration (Bertin Pharma, Bretonneux, France) according to the manufacturer’s instructions using a commercial ELISA kit.

### Histology examination

All kidney tissues were cleared by systemic perfusion with phosphate buffered saline (PBS) and fixed in 10% formalin. Paraffin-embedded kidney tissues were used for histologic analyses. A masson trichrome stain was performed to analyze the severity of the tubulointerstitial fibrosis. More than 20 randomly selected fields from the corticomedullary junction area were quantified, and the images were taken using a light microscope (Zeiss LSM 510, Carl Zeiss, Jena, Germany). The fibrotic area was quantified using MetaMorph imaging software (Molecular Devices Inc., Downingtown, PA) in selected fields. The ratio of the fibrotic area to the total selected field was expressed as the severity of tubulointerstitial fibrosis. For the assessment of mesangial widening, periodic acid-Schiff (PAS) staining was performed and mesangial matrix index was calculated. The mesangial matrix area and glomerular tuft area were quantified for each glomerular cross-section [30]. More than 30 glomeruli that were cut through the vascular pole were counted per kidney, and the average of measured areas was used for analysis. The mesangial matrix index was defined as the proportion of the glomerular tuft that was occupied by the mesangial matrix area (excluding nuclei). Mean glomerular tuft volume was calculated from the mean glomerular cross-sectional tuft area by Weibel’s method (glomerular tuft volume = glomerular cross-sectional tuft area^1.5^ × 1.38 / 1.05) [31].

Immunohistochemistry for ED-1, 8-hydroxy-2-deoxy guanosine (8-OHdG), and SGLT2 (Abcam) was performed. Briefly, after deparaffinization and hydration, the kidney sections were incubated in 0.5% Triton X-100–PBS solution and washed with PBS. Normal horse serum (Vector Laboratories, Inc., CA) was used for blocking non-specific antibody binding reaction. The sections were incubated overnight in a humidified chamber at 4°C with a primary antibody for ED-1 (Abcam, Cambridge, UK), 8-OHdG (Abcam), or SGLT2 (Abcam). Peroxidase-conjugated anti-mouse IgG (Vector Laboratories) was used as a secondary antibody. The sections were developed with a mixture of 0.05% 3,3′-diaminobenzidine containing 0.01% H_2_O_2,_ and analyzed under light microscopy (Olympus BX-50; Olympus Optical, Tokyo, Japan). Twenty high-power fields that included the renal corticomedullary junction were randomly selected in each section under x 200 magnification, and ED-1 or 8-OHdG-positive cells were counted. All of these sections were examined in a blind manner.

### Measurement of lipid peroxidation and hydrogen peroxide in the kidney tissues

For the evaluation of the lipid peroxidation level in the kidney tissues, malondialdehyde (MDA) concentration, a marker of free radical-mediated oxidative stress, was determined using thiobarbituric acid–trichloroacetic acid–HCl solution (0.375% thiobarbituric acid and trichloroacetic acid in 0.25 N HCl, pH 2.0) as described previously [32]. In brief, the kidney tissues were homogenized in a sucrose solution (70 mM sucrose, 210 mM mannitol, 1 mM EDTA, 10 mM Hepes in distilled water). After protein concentration was measured, 100 μg of lysates were added in a 1 ml of thiobarbituric acid-trichloroacetic acid-HCl solution (0.375% thiobarbituric acid, trichloroacetic acid in 0.25 N HCl, pH 2.0), and boiled at 100°C for 15 minutes. Absorbance was determined at a wavelength of 535 nm. The hydrogen peroxide (H_2_O_2_) concentration in kidney tissues was also measured using Fox reagent (0.25 M H_2_SO_4_, 1 M sorbitol, 25 mM ferrous ammonium sulfate, and 1 mM xylenol orange in distilled water) as described previously. H_2_O_2_ oxidizes iron (II) to iron (III), mediated by the catalytic action of sorbitol in the Fox reagent. Then, iron (III) forms a purple complex with xylenol orange. The absorbance was measured at a wavelength of 560 nm.

### Quantitative determination of the tissue collagen content in the kidney tissue

The total collagen amount in the kidney tissue was measured by acid hydrolysis of the kidney tissue section as described previously [33]. Briefly, each kidney tissue was weighed, hydrolysed in 6 N HCl for 18 hours at 110°C, and thoroughly dried at 75°C. After drying, samples were solubilized in a citric acid collagen buffer (0.23 mol/L citric acid, 0.88 mol/L sodium acetate trihydrate, 0.85 mol/L sodium hydroxide, and 1.2% acetic acid) and filtered through a 0.45 μm centrifugal filter unit (Ultrafree-MC, Millipore, Billerica, MA). Samples were diluted in the collagen buffer and then loaded into each well of a microplate, to which 100 μL of chloramine-T solution (1.4% chloramine-T and 10% n-propanol in citric acid buffer) was added to start the oxidation reaction. The microplate was incubated for 15 min at room temperature, and 100 μL of Ehrlich’s reagent (15% 4-dimethylamino-benzaldehyde, 62% n-propanol, and 18% perchloric acid) was added to each well to start the color reaction. The microplate was incubated in a large water bath for 20 min at 65°C. The amount of hydroxyproline was measured by a spectrophotometric assay at 550 nm. Total collagen in the kidney tissue was calculated on the assumption that collagen contains 12.7% hydroxyproline by weight.

### Semiquantitative immunoblotting

The proteins of the kidney tissues were extracted using a Pro-Prep Protein Extraction Kit (Intron Biotechnology, Inc., Seongnam, Korea) according to the manufacturer’s instructions. The protein concentration was measured using a Bradford assay (Bio-Rad Laboratories, Inc., Hercules, CA). Equal kidney protein samples were separated by 12 or 15% SDS-PAGE and transferred to nitrocellulose membranes. For immunodetection, the blots were incubated overnight at 4°C in PBS containing 0.1% Tween-20 and 5% skim milk with primary antibodies raised against the following proteins: AT1R (Santa Cruz Biotechnology, Santa Cruz, CA), Copper/zinc superoxide dismutase (Cu/ZnSOD) (Enzo Life Sciences, Inc., NY), Manganese-superoxide dismutase (MnSOD), catalase, type IV collagen, and SGLT2 (all from Abcam); β-actin (Sigma-Aldrich); The second antibody was HRP-linked anti-Rabbit IgG (Cell Signaling Technology, Beverly, MA) for AT1R, Cu/ZnSOD, SGLT2, and HPR-linked anti-Mouse IgG (Cell Signaling Technology) for MnSOD and β-actin. The protein bands were detected using a chemiluminescence imaging Systems (Fusion SL4-3500, Viber-Lourmat, France), and band densities were measured by Quantity One software (Bio-Rad).

### Statistical analysis

Values were expressed as means ± SE. Statistical differences among groups were calculated using a one-way ANOVA, followed by the Bonferroni test using the SPSS program (ver. 13.0). *P* < 0.05 was considered to be statistically significant.

## Results

### Physical and biochemical characteristics

**Fig 1:**
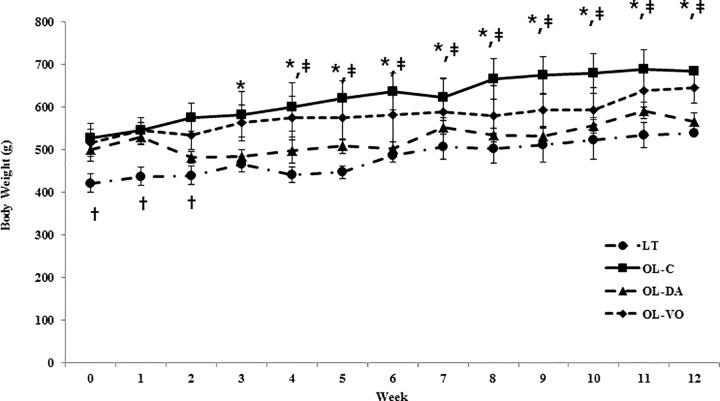
Changes in body weight during the experimental period in LETO (LT) and OLETF rats with saline (OL-C), dapagliflozine (OL-DA) or voglibose (OL-VO) treatment. *P < 0.05, OL-C *vs*. other groups; †*P* < 0.05, LT *vs*. other groups; ‡*P* < 0.05, OL-DA *vs*. OL-VO. Values are expressed as means ± SE.

**Fig 2:**
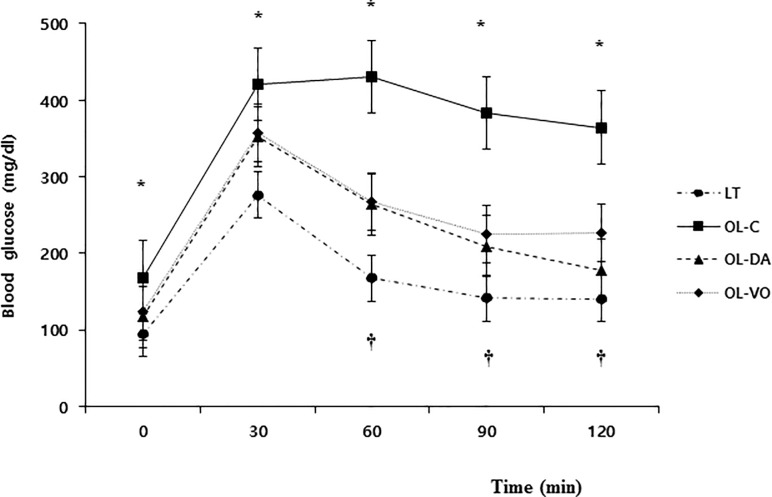
Intraperitoneal glucose tolerance test at 12 weeks in LETO (LT) and OLETF rats with saline (OL-C), dapagliflozin (OL-DA) or voglibose (OL-VO) treatment. *P < 0.05, OL-C vs. other groups; †*P* < 0.05, LT vs. other groups. Values are expressed as means ± SE.

The body weights of OL-C, OL-DA and OL-VO rats were heavier than those of LT rats at the early time of the experiment (*P* < 0.05, Fig 1). From 4 weeks of treatment (at age of 18 weeks), OL-C and OL-VO rats gained more body weight than LT and OL-DA rats, and these weight gains were maintained until 12 weeks of treatment was completed. At 12 weeks of treatment, the OL-C gained body weight significantly compared to other groups, and OL-VO rats weighed more than LT and OL-DA rats (*P* < 0.05, Fig 1, Table 1). Fasting blood glucose levels in the OL-C were significantly higher than in any other group, and those in OL-DA and OL-VO showed higher levels than those in LT (*P* < 0.05, Table 1). In the results of HbA1c and the intraperitoneal glucose tolerance test, the OL-C group had a higher increase in the glucose levels from 30 to 120 min (*P* < 0.05) than the other groups, and OL-DA and OL-VO showed higher blood glucose levels than that of LT from 60 to 120 min (*P* < 0.05, Fig 2). There were no significant differences in glucose and HbA1c levels between OL-DA and OL-VO groups. Serum sodium and potassium levels showed no significant differences among all groups, however, serum creatinine was significantly increased in OL-C (*P* < 0.05, Table 1). The 24-hr urine volume and urine glucose were significantly higher in the OL-DA than in any other group (*P* < 0.05, Table 1). On the other hand, the urine microalbumin and urine albumin to creatinine ratio (ACR) were markedly increased in the OL-C as compared to the other groups, and those in OL-DA and OL-VO were observed to be higher than in LT (*P* < 0.05). The creatinine clearance demonstrated the lowest values in the OL-C. Remarkably, urine microalbumin, ACR levels showed lower values in OL-DA than in the OL-VO group (*P* < 0.05, Table 1).

**Table 1:** Biochemical characteristics after 12 weeks of treatment.

Parameters	LT	OL-C	OL-DA	OL-VO
Body weight (g)	538.7 ± 47.1	684.3 ± 28.6*	566.3 ± 19.8	645.1 ± 36.9†,‡
Fasting blood glucose (mg/dl)	94.7 ± 1.2	168.6 ± 17.9*	116.6 ± 1.7†	123.3 ± 6.2†
HbA1c (%)	3.71 ± 0.1	5.81 ± 1.7*	3.77 ± 0.1	3.93 ± 0.4
Serum sodium (mEq/L)	147.2 ± 1.1	143.2 ± 0.8	143.6 ± 0.3	143.6 ± 0.4
Serum potassium (mEq/L)	5.92 ± 0.5	5.35 ± 0.2	5.65 ± 0.4	5.19 ± 0.4
Serum creatinine (mg/dl)	0.58 ± 0.02	1.21 ± 0.3*	0.34 ± 0.02	0.53 ± 0.1
24hr urine volume (ml)	8.9 ± 3.3	11.6 ± 4.1	24.6 ± 5.4*	12.5 ± 3.1
Urine glucose (mg/dl)	50.7 ± 11.2	64.3 ± 11.0	9100 ± 443.2*	31.5 ± 9.3
Urine microalbumin (μg/ml)	14.9 ± 3.7	127.2 ± 23.1*	47.9 ± 3.6†	88.9 ± 12.3†,‡
Urine albumin/creatinine (μg/g)	0.99 ± 0.3	12.3 ± 2.0*	4.86 ± 0.5†	6.68 ± 0.9†,‡
Creatinine clearance (ml/min)	1.89 ± 0.4	0.86 ± 0.2*	3.17 ± 0.7†	2.07 ± 0.9‡

Values are means ± SD. LT, LETO; OL-C, OLETF-control; OL-DA, OLETF-dapagliflozin; OL-VO, OLETF-voglibose.

* *P* < 0.05, OL-C vs. other groups

† *P* < 0.05, OL-DA and OL-VO vs. LT

‡ *P* < 0.05, OL-VO vs. OL-DA.

### Plasma renin activity, serum aldosterone, and intrarenal RAS expressions

**Fig 3:**
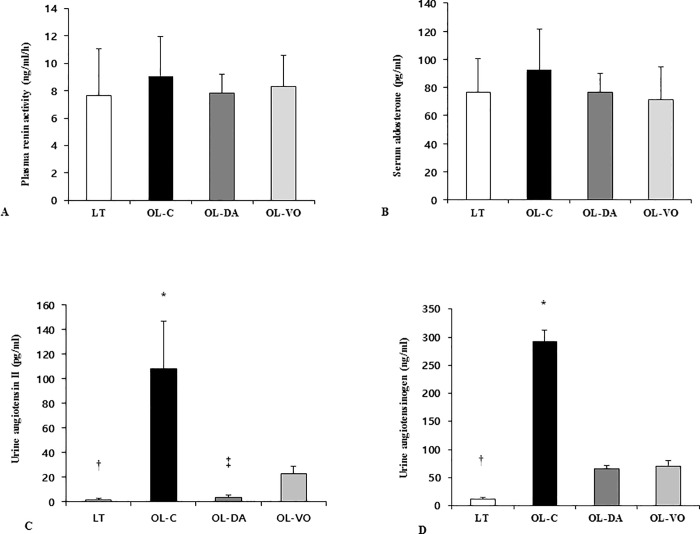
Systemic and intrarenal RAS activation at 12 weeks. Plasma renin activity (A), serum aldosterone (B), urinary angiotensin II (C) and angiotensinogen (D) in LETO (LT) and OLETF rats with saline (OL-C), dapagliflozin (OL-DA) or voglibose (OL-VO) treatment. *P < 0.05, OL-C vs. other groups; †*P* < 0.05, LT vs. other groups; ‡*P* < 0.05, OL-DA vs. OL-VO. Values are expressed as means ± SE.

**Fig 4:**
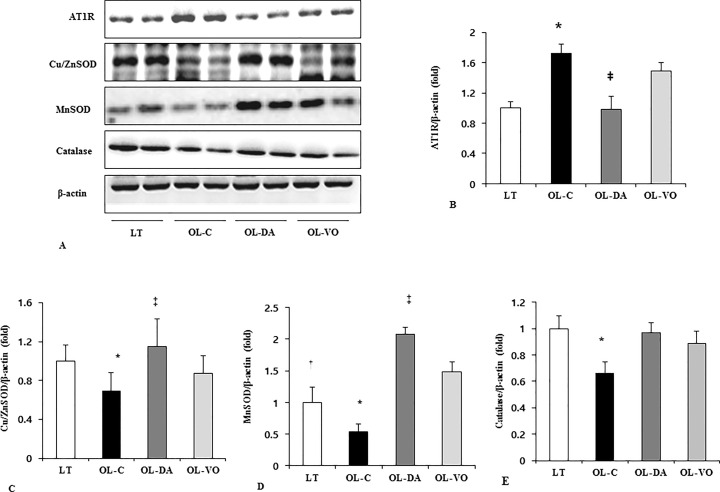
The expressions of RAS and antioxidant enzymes in renal tissues. (A) Representative immunoblots of AT1R, Cu/ZnSOD, MnSOD and catalase. Quantitative analyses of the expression of AT1R (B), Cu/ZnSOD (C), MnSOD (D), catalase (E) in LETO (LT) and OLETF rats with saline (OL-C), dapagliflozine (OL-DA) or voglibose (OL-VO) treatment. *P < 0.05, OL-C vs. other groups; †*P* < 0.05, LT vs. other groups; ‡*P* < 0.05, OL-DA vs. OL-VO. Values are expressed as means ± SE. AT1R, Ang II type I receptor

There were no differences in plasma renin activity or serum aldosterone level among the groups (Renin activity, LT, 76.8 ± 23.7, OL-C, 92.7 ± 28.6, OL-DA, 76.3 ± 16.5 and OL-VO, 71.5 ± 23.1 ng/ml/h, respectively, *P* > 0.05; serum aldosterone, LT, 7.7 ± 3.4, OL-C, 9.1 ± 2.9, OL-DA, 7.9 ± 1.4 and OL-VO, 8.3 ± 2.3 pg/ml, respectively, *P* > 0.05; Fig 3A and 3B). However, 24-h urinary Ang II and AGT level were increased in the OL-C group compared to those in other groups (Ang II, OL-C *vs*. LT, OL-DA and OL-VO, 108.1 ± 38.8 *vs*. 1.6 ± 1.0, 3.2 ± 1.7 and 22.7 ± 61.5 pg/ml, respectively, *P* < 0.05; AGT, OL-C *vs*. LT, OL-DA and OL-VO, 292.8 ± 19.5 *vs*. 11.95 ± 2.1, 65.2 ± 6.6 and 70.3 ± 10.6 ng/ml, respectively, *P* < 0.05; Fig 3C and 3D). Remarkably, urine Ang II levels were significantly different between OL-DA and OL-VO groups (Fig 3C). The immunoblot demonstrated that the renal cortical AT1R expression in the OL-C was significantly higher compared with the other groups (Fig 4A). In contrast to the OL-C, the dapagliflozin and voglibose treatments resulted in a remarkable decrease in the expression of AT1R (*P* < 0.05, Fig 4A and 4B). Furthermore, AT1R expression was significantly attenuated in OL-DA compared to the OL-VO group (Fig 4B).

### Oxidative stress in renal tissues

To examine the changes of oxidative stress associated RAS activation and the effect of dapagliflozin on the suppression of oxidative stress in renal tissues, immunoblot analyses for antioxidant enzymes, including Cu/ZnSOD, MnSOD, and catalase, were performed. As shown in Fig 4, the expressions of Cu/ZnSOD, MnSOD, and catalase were decreased in the OL-C. In contrast, treatment with dapagliflozin resulted in a significantly greater increase in the expression of Cu/ZnSOD and MnSOD in the OL-DA compared to those in OL-C and OL-VO (*P* < 0.05, respectively, Fig 4C and 4D). The expression of catalase, an H_2_O_2_-inducible anti-oxidant enzyme, was significantly decreased in OL-C, and dapagliflozin treatment restored its expression (OL-C *vs*. LT and OL-DA, *P* < 0.05, respectively, Fig 4E). Taken together, these findings suggested that oxidative stress in the OL-C rats could be ameliorated by dapagliflozin and voglibose treatment. Interestingly, Cu/ZnSOD and MnSOD expressions were more increased in OL-DA rats compared to those in OL-VO group (Fig 4).

**Fig 5:**
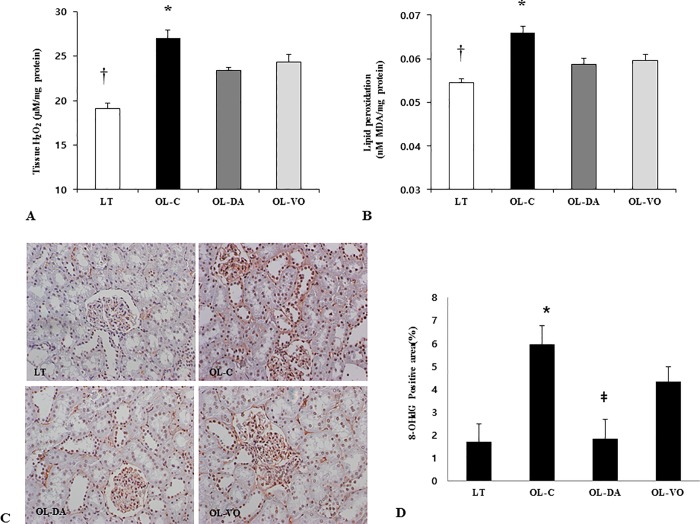
Oxidative stress markers in renal tissues. The levels of renal H_2_O_2_ (A) and malondialdehyde (MDA) (B), 8-OHdG immunostaining (x 200, C), quantitative measurement of 8-OHdG staining (D) in LETO (LT) and OLETF rats with saline (OL-C), dapagliflozin (OL-DA) or voglibose (OL-VO) treatment. *P < 0.05, OL-C vs. other groups; †*P* < 0.05, LT vs. other groups; ‡*P* < 0.05, OL-DA vs. OL-VO. Values are expressed as means ± SE. 8-OHdG, 8-hydroxy-2-deoxy guanosine.

Oxidative stress in the kidney was also measured by the assessment of the renal tissue levels of H_2_O_2_ and lipid peroxidation. Direct measurement of the tissue levels of H_2_O_2_ and MDA, a marker of free radical-mediated oxidative stress in the kidneys of OL-C rats, showed higher values than any other group, which were significantly decreased by treatment with dapagliflozin or voglibose (H_2_O_2_, OL-C *vs*. LT, OL-DA and OL-VO, 27.1 ± 0.9 *vs*. 19.1 ± 0.6, 23.4 ± 0.3 and 24.3 ± 0.9 μg/mg protein, respectively, *P* < 0.05, Fig 5A; MDA, OL-C *vs*. LT, OL-DA and OL-VO, 0.07 ± 0.001 *vs*. 0.065 ± 0.001, 0.06 ± 0.002 and 0.06 ± 0.001 nM MDA/mg protein, respectively, *P* < 0.05; Fig 5B). Remarkably, both oxidative stress markers were more decreased in OL-DA rats compared to those in OL-VO groups (Fig 5A and 5B). In addition, dapagliflozin or voglibose treatment significantly attenuated the renal immunostaining of 8-OHdG than that of OLETF control groups (Fig 5C). The relative percentage of 8-OHdG-positive nuclei was also significantly decreased in the OL-DA group compared to that in the OL-VO group (Fig 5D).

### Renal inflammatory cell infiltration, extracellular matrix accumulation, and fibrosis

**Fig 6:**
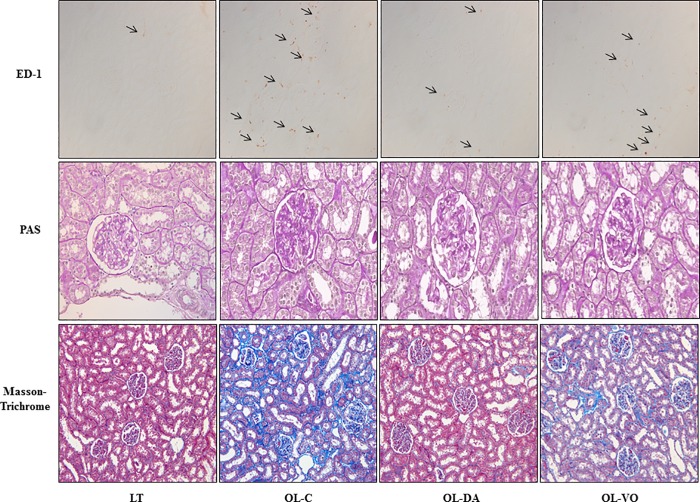
Changes in glomerular or tubulointerstitial phenotypes in LETO or OLETF rats. Representative sections for assessing the inflammatory cell infiltration (immunostaining for ED-1, x 200), glomerular expansion (PAS, x 400) and tubulointerstitial fibrosis (Masson trichrome, x 200) in LETO (LT) and OLETF rats with saline (OL-C), dapagliflozine (OL-DA) or voglibose (OL-VO) treatment.

**Fig 7:**
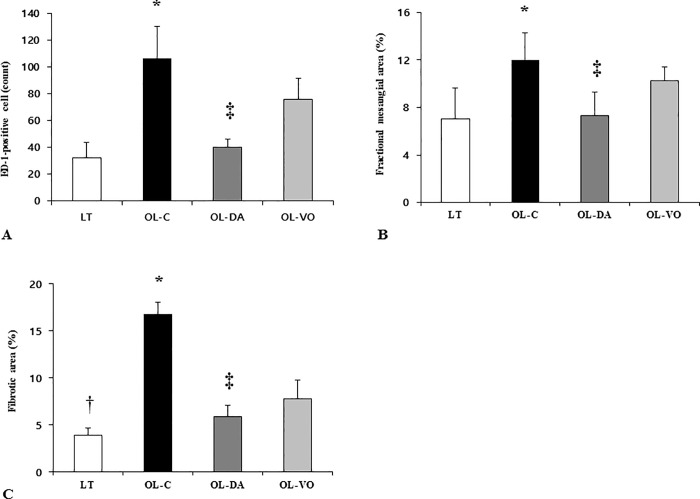
(A) Semi-quantitative analysis of the number of ED-1 positive cells, (B) fractional mesangial area, and (C) fibrotic area in kidney tissue. *P < 0.05, OL-C vs. other groups; †*P* < 0.05, LT vs. other groups; ‡*P* < 0.05, OL-DA vs. OL-VO. Values are expressed as means ± SE.

Staining for ED-1 showed the degree of interstitial macrophage infiltration (Fig 6). Immunohistochemical analysis for ED-1 indicated that the number of ED-1 positive cells markedly increased in the periglomerular and interstitial area of the OL-C (106.4 ± 23.9 cells) compared to the LT (31.9 ± 11.6 cells) (OL-C *vs*. LT, *P* < 0.05). Dapagliflozin significantly decreased the infiltration of ED-1 positive cells in OL-DA (40.0 ± 6.1 cells) (OL-C *vs*. OL-DA, *P* < 0.05, Fig 7A). In contrast, the treatment of voglibose did not decrease the number of ED-1 positive cells in the kidney of the OL-VO (31.9 ± 11.6 cells) (OL-VO *vs*. LT and OL-DA, *P* < 0.05, respectively).

Mesangial expansion is one of the characteristic findings of diabetic nephropathy [5]. Analysis of PAS-stained kidney sections revealed that there were no differences in the fractional mesangial areas between the LT and OL-DA rats (Fig 6). In contrast, there was a marked increase in the mesangial area in the OL-C compared with the LT (*P* < 0.05). These changes of mesangial expansion were ameliorated in dapagliflozin-treated OL-DA rats (*P* < 0.05, Figs 6 and 7B). In Masson trichrome staining (Fig 6), extracellular matrix deposition within the renal tubulointerstitial area was increased in the kidney of OL-C compared to that of LT (*P* < 0.05). Dapagliflozin treatment significantly ameliorated tubulointerstitial fibrosis in the kidney of OL-DA (*P* < 0.05, Fig 7C). Interestingly, there were significant differences in mesangial expansion and fibrotic area between OL-DA and OL-VO groups (Fig 7B and 7C).

**Fig 8:**
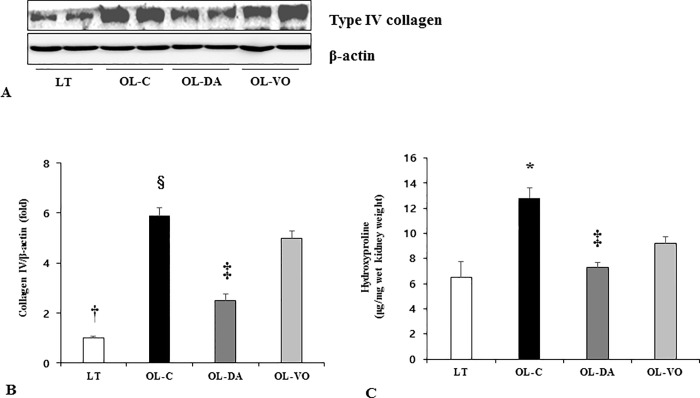
The expression of type IV collagen and total collagen content in renal tissues. (A) Representative immunoblot of type IV collagen, (B) quantitative analyses of the expression of type IV collagen and (C) the amount of hydroxyproline in renal tissue in LETO (LT) and OLETF rats with saline (OL-C), dapagliflozin (OL-DA) or voglibose (OL-VO) treatment. *P < 0.05, OL-C vs. other groups; †*P* < 0.05, LT vs. other groups; ‡*P* < 0.05, OL-DA vs. OL-VO; §*P* < 0.05, OL-C vs. OL-DA. Values are expressed as means ± SE.

SGLT2 staining in renal tissue was significantly increased in OL-C groups compared to those of LT rats. But, after dapagliflozin treatment, its staining was significantly attenuated (S1 Fig). In immunoblot analysis, the expression of type IV collagen showed a similar pattern to the findings of extracellular matrix deposition. It was prominently increased in OL-C, the degree of which was reversed by dapagliflozin treatment in OL-DA (*P* < 0.05, Fig 7A and 7B). In addition, the collagen type IV expression level was significantly higher in the OL-VO group compared to in the OL-DA group (Fig 8A and 8B). The amount of hydroxyproline in the kidneys, reflecting total collagen content in renal tissue was found to be increased in the OL-C. This diabetes-associated increase of the hydroxyproline level seen in OL-C rats was significantly reversed by dapagliflozin and voglibose treatment (Fig 8C). The hydroxyproline level in OL-DA tended to be less, in comparison to OL-VO with statistical significance (LT, OL-C, OL-DA and OL-VO, 6.5 ± 1.3, 10.8 ± 1.1, 7.8 ± 0.5 and 9.0 ± 0.7 hydroxyproline μg/mg wet kidney weight, respectively; OL-C *vs*. LT, OL-DA and OL-VO, *P* < 0.05; OL-DA *vs*. OL-VO, *P* < 0.05) (Fig 8C).

Accordingly, SGLT2 expression in Western blot analysis was significantly decreased in the OL-DA group compared to that of OL-C rats (S2A Fig). In addition, dapagliflozin treatment also attenuated SGLT2 expression compared to that in the voglibose-treated OLETF group (S2B Fig).

## Discussion

We evaluated the effect of dapagliflozin, an SGLT2 inhibitor, in renal damage using genetically modified animal model of type 2 diabetes, OLETF rats. The present study demonstrated renal injury in OLETF rats as evidenced by decreased renal function, renal inflammation and fibrosis, as well as RAS activation and decreased expressions of antioxidant enzymes. However, long term treatment with dapagliflozin revealed that the beneficial effects were not only confined to its glucose-lowering actions, but also its ability to reduce RAS activation, oxidative stress, renal inflammation and fibrosis, which are main causes of the progression of diabetic renal disease. These results demonstrated that administration of dapagliflozin ameliorated renal RAS activation and decreased oxidative stress production, proinflammatory cell infiltration in the kidney, and attenuated mesangial matrix expansion and interstitial fibrosis in OLETF rats. We also compared the renal effects of two antidiabetic medications, dapagliflozin (SGLT2 inhibitor) and voglibose (alpha-glucosidase inhibitor) with different glucose-lowering mechanisms in this experiment. Voglibose inhibits intestinal alpha-glucosidase activity and generally attenuates hyperglycemia by lowering postprandial hyperglycemia. In this study, glycemic status was not different between OL-DA and OL-VO rats throughout the observation period. Despite similar glucose-lowering actions, dapagliflozin showed more decrease in renal RAS and more increase in antioxidant enzyme expressions in diabetic kidneys than voglibose treatment. Therefore, we suggest that dapagliflozin may provide additional benefits on the progression or prevention of diabetic nephropathy in type 2 diabetes.

Local activation of the intrarenal RAS is considered as fundamental to the onset of diabetic nephropathy [11]. In addition, it is well-established that RAS plays an important role in the development of renal fibrosis and following ESRD [11, 34]. Preclinical studies demonstrated that intrarenal RAS in individuals with diabetes was inappropriately activated, leading to local Ang II overproduction in proximal tubular cells, mesangial cells, and podocyte, although the systemic RAS was generally suppressed [12–14, 35]. Ang II is also known to be the generator of oxidative stress in renal mesangial cells and to inhibit the basal protein expression of Nrf2 and its downstream antioxidant enzymes [36]. It has been reported that kidney Ang II levels, urinary AGT, and mRNA of ANG and renin levels were higher in OLETF than in LETO rats, even in a prediabetic stage [15]. In type 2 diabetes, the main tubulointerstitial changes seen are those of proximal tubular cell hyperplasia and hypertrophy in early diabetes, followed by atrophy and interstitial fibrosis as the condition progresses [37]. Type IV collagen, a marker of renal extracellular matrix production and renal fibrosis are known as characteristic features of advanced diabetic nephropathy, and local Ang II activation is a key factor for their progression [37, 38].

The mechanisms by which high glucose stimulates angiotensinogen expression in proximal tubular cells (PTCs) have been investigated. Previously, high glucose stimulated AGT gene expression in rat immortalized PTCs, mediated in part via PKC and p38 MAPK signal pathway [39] and activation of the hexosamine biosynthesis pathway [40]. The possible mechanism of stimulatory action of high glucose on ANG gene expression (downstream pathway after p38 MAPK and PKC activation) in PTC was that high glucose induced cAMP response element-binding protein (CREB) phosphorylation via the PKC signaling pathway [41], leading to interact with phosphorylated nuclear ATF-2 to form a heterodimer that binds to the cAMP-responsive element in the 5'-flanking region of the rat ANG gene [42]. In addition, Ang II mediates glucose-induced upstream stimulatory factor 2 (USF2) expression in renal PTCs through AT1 receptor-dependent activation of the transcription factor CREB [43].

SGLT2 inhibitors are a novel new class of antidiabetic treatment for type 2 diabetes. They improve insulin sensitivity, decrease fat mass with regional adipose tissue distribution, promote weight loss, and lower blood pressure in patients with type 2 diabetes by blocking glucose reabsorption in the kidney [44–45]. However, the influence of SGLT2 inhibitors on diabetic nephropathy and renal dysfunction has not been clarified. Renal PTCs produce inflammatory molecules and growth factors in response to high glucose [40, 46]. Therefore, a reduction in glucose transit through the PTCs may attenuate PTC-induced inflammation and fibrosis in diabetic nephropathy. Terami et al. has shown that dapagliflozin decreased macrophage infiltration into kidney and diabetes-induced oxidative stress in a dose-dependent manner in db/db mice [47]. Nagata et al. also demonstrated that tofogliflozin reduced albuminuria and glomerular hypertrophy in db/db mice [48]. Ipragliflozin administration in type 2 diabetic mice showed decreased creatinine clearance and urinary ACR [49].

Our study provides and *in vivo* evidence that SGLT2 inhibition attenuated diabetic nephropathy (microalbuminuria), inflammatory and fibrotic markers. We demonstrated that the renal expression of AT1R was increased in OL-C rats, and that treatment with dapagliflozin led to the down regulation of AT1R. Dapagliflozin also significantly increased the expression levels of the antioxidant enzymes, including Cu/ZnSOD, MnSOD, and catalase in diabetic nephropathy. These findings suggest that SGLT2 inhibitors might have additional beneficial influences on a diabetic kidney, beyond glucose-lowering effects.

In conclusion, progressive renal inflammation and fibrosis in the OLETF rats was associated with the upregulation of oxidative stress and renal RAS activation. Treatment with dapagliflozin prevented the development of nephropathy by reducing oxidative stress from an increasing production of endogenous antioxidant enzymes and fibrotic markers in renal tissues. Therefore, SGLT2 inhibition could attenuate PTCs glucotoxicity and renal inflammation by suppressing RAS activation. Beyond the glucose-lowering effect, dapagliflozin might be a promising therapeutic approach for preventing and improving diabetic nephropathy in type 2 diabetes. In the future, further clinical studies are needed to determine the pharmacological mechanism of SGLT2 inhibition associated with activation of RAS in type 2 diabetes.

## Supporting Information

S1 Fig Representative sections for SGLT2 immunostaining (x 200) in LETO (LT) and OLETF rats with saline (OL-C), dapagliflozine (OL-DA) or voglibose (OL-VO) treatment (A). Quantitative analysis of the proportion of SGLT2 positive cells in renal tissue (B). *P < 0.05, OL-C vs. other groups; †*P* < 0.05, LT vs. other groups; ‡*P* < 0.05, OL-DA vs. OL-VO. Values are expressed as means ± SE.
(TIF)

S2 Fig Representative Western blot analysis of SGLT2 in renal tissues (A). Quantitative analysis of SGLT2 expression in OL-C group was significantly different from that in OL-DA group (B). *P < 0.05, OL-C vs. other groups; ‡*P* < 0.05, OL-DA vs. OL-VO. Values are expressed as means ± SE.
(TIF)

## Abbreviations

SGLT2: Sodium-glucose Co-transporter 2
RAS: Renin-angiotensin system
ESRD: End stage renal disease
Ang II: Angiotensin II
AGT: Angiotensinogen
AT1R: Angiotensin type 1 receptor
AT2R: Angiotensin type 2 receptor
HbA1c: Hemoglobin A1c
8-OHdG: 8-hydroxy-2-deoxy guanosine
MDA: Malondialdehyde
Cu/ZnSOD: Copper/zinc-superoxide dismutase
MnSOD: Manganese-superoxide dismutase
ACR: Urine albumin to creatinine ratio
PTCs: Proximal tubular cells
