# Supplementary figures and images for: Effect of Sodium-Glucose Co-Transporter 2 Inhibitor, Dapagliflozin, on Renal Renin-Angiotensin System in an Animal Model of Type 2 Diabetes
Source: PLoS One. 2016 Nov 1;11(11):e0165703. doi: 10.1371/journal.pone.0165703 (PMC5089752; doi:10.1371/journal.pone.0165703)

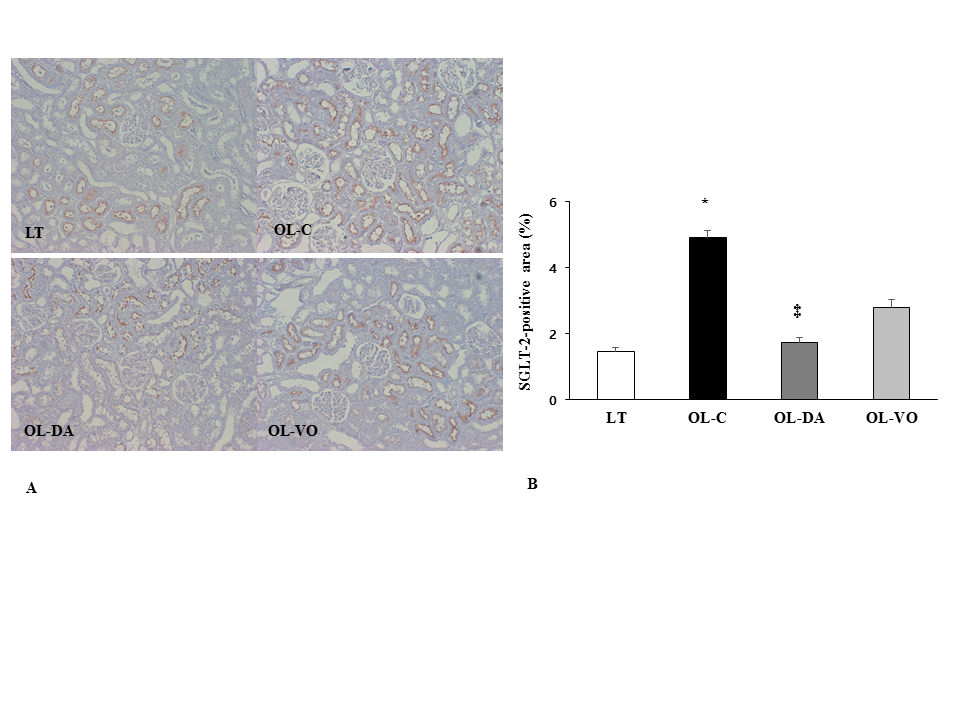

Supplement: S1 Fig — Representative sections for SGLT2 immunostaining (x 200) in LETO (LT) and OLETF rats with saline (OL-C), dapagliflozine (OL-DA) or voglibose (OL-VO) treatment (A). Quantitative analysis of the proportion of SGLT2 positive cells in renal tissue (B). *P < 0.05, OL-C vs. other groups; †P < 0.05, LT vs. other groups; ‡P < 0.05, OL-DA vs. OL-VO. Values are expressed as means ± SE. (TIF) [file pone.0165703.s001.tif]

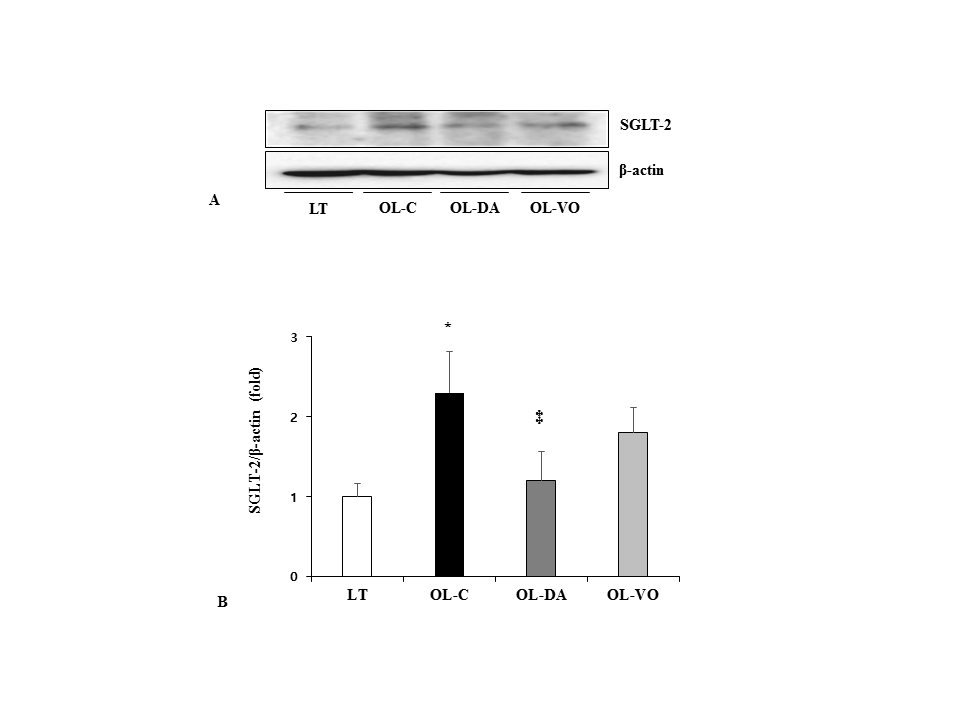

Supplement: S2 Fig — Representative Western blot analysis of SGLT2 in renal tissues (A). Quantitative analysis of SGLT2 expression in OL-C group was significantly different from that in OL-DA group (B). *P < 0.05, OL-C vs. other groups; ‡P < 0.05, OL-DA vs. OL-VO. Values are expressed as means ± SE. (TIF) [file pone.0165703.s002.tif]
